# Reduced intensity conditioning increases risk of severe cGVHD: identification of risk factors for cGVHD in a multicenter setting

**DOI:** 10.1007/s12032-018-1127-2

**Published:** 2018-04-25

**Authors:** Gabriel Afram, Jose Antonio Pérez Simón, Mats Remberger, Teresa Caballero-Velázquez, Rodrigo Martino, Jose Luis Piñana, Olle Ringden, Albert Esquirol, Lucia Lopez-Corral, Irene Garcia, Oriana López-Godino, Jordi Sierra, Dolores Caballero, Per Ljungman, Lourdes Vazquez, Hans Hägglund

**Affiliations:** 1 Department of Hematology, Karolinska University Hospital Huddinge, Stockholm, Sweden; 2 Department of Hematology, Instituto de Biomedicina de Sevilla (IBIS), Hospital Universitario Virgen del Rocío/CSIC/Universidad de Sevilla, Seville, Spain; 3 Centre for Allogeneic Stem Cell Transplantation, Karolinska University Hospital Huddinge, Stockholm, Sweden; 4 Department of Hematology, Hospital de la Santa Creu i Sant Pau, Barcelona, Spain; 5 Department of Hematology, Hospital Universitario de Salamanca-IBSAL, Salamanca, Spain; 6 Department of Hematology, Hospital Clinico Universitario, Valencia, Spain; 7 Division of Hematology, Department of Medical Sciences, Uppsala University Hospital, Uppsala, Sweden

**Keywords:** ATG, Graft-versus-host disease (GVHD), Risk factor

## Abstract

Chronic graft-versus-host disease (cGVHD) remains a major cause of morbidity and mortality after allogeneic hematopoietic stem cell transplantation (HSCT). Aim is to identify risk factors for the development of cGVHD in a multicenter setting. Patients transplanted between 2000 and 2006 were analyzed (*n* = 820). Donors were HLA-identical siblings (57%), matched unrelated donors (30%), and HLA-A, B or DR antigen mismatched (13%). Reduced intensity conditioning (RIC) was given to 65% of patients. Overall incidence of cGVHD was 46% for patients surviving more than 100 days after HSCT (*n* = 747). Older patient age [HR 1.15, *p* < 0.001], prior acute GVHD [1.30, *p* = 0.024], and RIC [1.36, *p* = 0.028] increased overall cGVHD. In addition, RIC [4.85, *p* < 0.001], prior aGVHD [2.14, *p* = 0.001] and female donor to male recipient [1.80, *p* = 0.008] increased the risk of severe cGVHD. ATG had a protective effect for both overall [0.41, *p* < 0.001] and severe cGVHD [0.20, *p* < 0.001]. Relapse-free survival (RFS) was impaired in patients with severe cGVHD. RIC, prior aGVHD, and female-to-male donation increase the risk of severe cGVHD. ATG reduces the risk of all grades of cGVHD without hampering RFS. GVHD prophylaxis may be tailored according to the risk profile of patients.

## Introduction

Chronic graft-versus-host disease (cGVHD) remains one of the most severe complications after allogeneic hematopoietic stem cell transplantation (HSCT), affecting both the quality of life and mortality of long-term survivors [1–4]. Its impact on morbidity and mortality varies depending on the severity and number of organs involved, allowing the classification of patients into mild, moderate, and severe cGVHD according to the NIH, and identifying those at low, intermediate, or high risk of developing GVHD-related morbidity and mortality. Chronic GVHD is associated with a graft-versus-tumor effect (GVT) that decreases the risk of relapse after transplant [5]. These findings emphasize the importance of appropriate management of cGVHD, which should be individualized according to the patients’ characteristics.

Risk factors for cGVHD include high recipient age, prior acute GVHD, female donor to male recipient [6], HLA disparity between recipient and donor, and use of peripheral blood as a source of progenitor cells [7–9]. CGVHD is an increasingly frequent complication after HSCT due, at least in part, to the more frequent use of peripheral blood stem cells, higher age of recipients/donors, and increased use of mismatched and unrelated donors. Recently, we retrospectively classified a large cohort of patients in terms of cGvHD subtype and severity according to the NIH proposal [10]. To the best of our knowledge, there are no data on cGvHD risk factor analysis based on the cGvHD NIH classification.

Various studies have attempted to identify the best strategy to prevent GVHD, and to date, only the use of in vitro or in vivo T cell depletion has been shown to reduce the risk of cGVHD, although its impact on survival has been relatively limited in unselected series of patients [11]. Therefore, the prophylaxis regimen should be tailored on the basis of individual patient and transplant characteristics, and more effective immunosuppressive strategies could benefit patients at high risk of severe forms of cGVHD and low risk of relapse, while the contrary could apply to patients at high risk of relapse in the event of displaying a lower risk of cGVHD.

Our study aimed to highlight risk factors for developing mild, moderate, and severe cGVHD in a multicenter setting. We retrospectively included 747 patients who had undergone HSCT between the years 2000–2006.

## Patients and methods

Eight hundred and twenty patients undergoing HSCT at three different centers from January 2000 to December 2006 were included in this retrospective study. The analysis was restricted to those patients surviving more than 100 days after HSCT (*n* = 747). The number of patients included from each center was: Karolinska (*n* = 425), Salamanca (*n* = 162) and Sant Pau (*n* = 160). The study protocol was approved by the regional ethics committees (Regionala Etikprövningsnämnden, Stockholm and Comité Etico CEIM (Comité de ética de la investigación con medicamentos) for Spain and was performed in accordance with the Declaration of Helsinki. Chronic GVHD was retrospectively categorized according to the NIH consensus criteria [12]. The patients’ characteristics are summarized in Table 1.

**Table 1:** Characteristics of HSCT patients with or without chronic GVHD

Factor	No cGVHD	cGVHD	*p* value
*N* =	409	338	
Age	37 (< 1–69)	51 (< 1–70)	< 0.001
Donor age	38 (0–74)	45 (0–77)	< 0.001
Sex (male/female)	241/168	214/124	0.25
Donor sex (male/female)	239/164	179/152	0.18
Female to male	76 (19%)	89 (26%)	0.013
Disease stage (early/late)	180/199	129/195	0.04
Stem cell source (PBSC/BM/CB)	278/108/23	290/43/5	< 0.001
CD34 + cell dose (× 10^6^/kg)	6.8 (0.1–68)	6.7 (0.1 to − 19.9)	0.89
*Donor*			
Sibling	196 (48%)	242 (72%)	< 0.001
MUD	153 (37%)	64 (19%)	
Mismatched	60 (15%)	31 (9%)	0.03
*Conditioning*			
MAC	165 (43%)	85 (26%)	< 0.001
RIC	232 (57%)	245 (74%)	
ATG	233 (57%)	92 (27%)	
*Diagnosis*			
Non-malignant	53 (13%)	17 (5%)	< 0.001
AML/ALL	90/52 (35%)	81/31 (33%)	Ns
CML/CLL	41/13 (13%)	36/21 (17%)	Ns
Lymphoma	55 (13%)	53 (16%)	Ns
MDS/MPS	36 (9%)	40 (12%)	Ns
Myeloma	29 (7%)	30 (9%)	Ns
Solid tumor	30 (7%)	13 (4%)	Ns
Other	10 (2%)	16 (5%)	Ns

*Early stage* CR1/CP1, *Late stage* beyond CR1/CP1, *PBSC* peripheral blood stem cells, *BM* bone marrow, *CB* cord blood, *MUD* matched unrelated donor, *MAC* myeloablative conditioning, *RIC* reduced intensity conditioning, *ATG* anti-thymocyte globulin, *AML* acute myeloid leukemia, *ALL* acute lymphoid leukemia, *CML* chronic myeloid leukemia, *CLL* chronic lymphoid leukemia, *MDS* myelodysplastic syndrome, *MPS* myeloproliferative syndrome

The median age at the time of transplantation was 44 years (< 1–70). The most common diagnosis was acute leukemia (acute myeloid leukemia or acute lymphoblastic leukemia) in 34% of patients; chronic leukemia was diagnosed in 15% and lymphoma in 14%.

### Donors and stem cell source

Fifty-seven percent of patients received sibling donor grafts, 13% received grafts with one HLA-A, B, or DR antigen or allele mismatch, and 30% received grafts from HLA-A, B, and DR-matched unrelated donors. All patients and donors were typed using PCR-SSP high-resolution typing for both HLA class I and II alleles [13]. Seventy-six percent received peripheral blood hematopoietic stem cells (PBSC) from G-CSF stimulated donors, 20% received bone marrow grafts, and 4% received cord blood grafts.

### Conditioning

Myeloablative regimens were used in 34% of cases, the rest receiving reduced intensity conditioning (RIC). Forty-four percent received in vivo T cell depletion with anti-thymocyte globulin (ATG, Thymoglobulin, Genzyme, Cambridge, MA, USA) (*n* = 295) or Campath^®^ (*n* = 30). Patients at Karolinska (*center A*) received ATG (Thymoglobulin, Genzyme, Cambridge, MA, USA) at a dose of 4–8 mg/kg. Patients from Salamanca and Sant Pau (*centers B and C*) received ATG at a dose of 7.5 mg/kg. ATG was administered for 3–4 days with the last dose given on day 1 or 2. ATG was administered to patients with unrelated donors, HLA-mismatched donors, and those with non-malignant diseases.

### GVHD prophylaxis and treatment

Most patients received GVHD prophylaxis with cyclosporine A or tacrolimus combined with methotrexate (80%), while 11% received mycophenolate mofetil instead of methotrexate. The remaining 9% of patients received CyA or tacrolimus combined with prednisone or other immunosuppressive regimes.

First-line treatment for cGVHD was based on cyclosporine A or tacrolimus plus prednisone. Disease response was generally evaluated 5 weeks after initiation of treatment and subsequently every 3 months until cessation of treatment.

All patients received antibacterial, antifungal, and antiviral prophylaxis according to standard protocols at each center.

### Statistical analysis

The incidence of chronic GVHD was estimated using an estimator of cumulative incidence curves. Patients were censored at the time of death or last follow-up. Only patients surviving more than 100 days after HSCT were included in the analysis and the competing event was death without chronic GVHD. Categorical parameters were compared using Chi-square test and continuous variables were compared using the Mann–Whitney test. Multivariate predictive analyses were performed using Gray’s test and the proportional sub-distribution hazard regression model of Fine and Gray [14]. A stepwise backward procedure was used to construct a set of independent predictors. All predictors with a *p* value below 0.10 in the univariate analysis were introduced into the multivariate model and sequentially removed if the *p* value was above 0.05. Risk factors included in the univariate analysis were: patient and donor age, patient and donor sex, sex mismatch, disease stage, stem cell source, donor type, conditioning, ATG, diagnosis, center, GVHD prophylaxis, CD34 + cell dose and prior aGVHD. All tests were two-sided. The type I error rate was fixed at 0.05 for factors potentially associated with time-to-event outcomes. Analyses were performed using the cmprsk package (developed by Gray, June 2001), S-PLUS 6.2 software and Statistica software.

## Results

The overall cumulative incidence of acute GVHD was 56% and that of cGVHD was 48% (95% CI 44–52%). The incidence of cGVHD at the three centers A, B, and C was 32% (27–37%), 70% (63–77%), and 58% (50–66%), respectively. The percentage of children transplanted at each center was 29, 6, and 0%, respectively, for center A, B, and C. Median time to onset for cGVHD was 5.7 (2.0–77) months post-transplant.

ATG was administered at a frequency of 71, 13, and 1% in the three centers, respectively. The percentage of sibling donor transplants at each center was 42, 79, and 81%, respectively. Results were similar in terms of survival at the three centers [53% (48–58%), 49% (41–57%), 52% (45–59%), respectively] as well as in terms of relapse-free survival (RFS) [46% (41–51%), 39% (31–47%), 42% (35–49%)] at 5 years.

### Risk factors for overall cGVHD

**Fig. 1:**
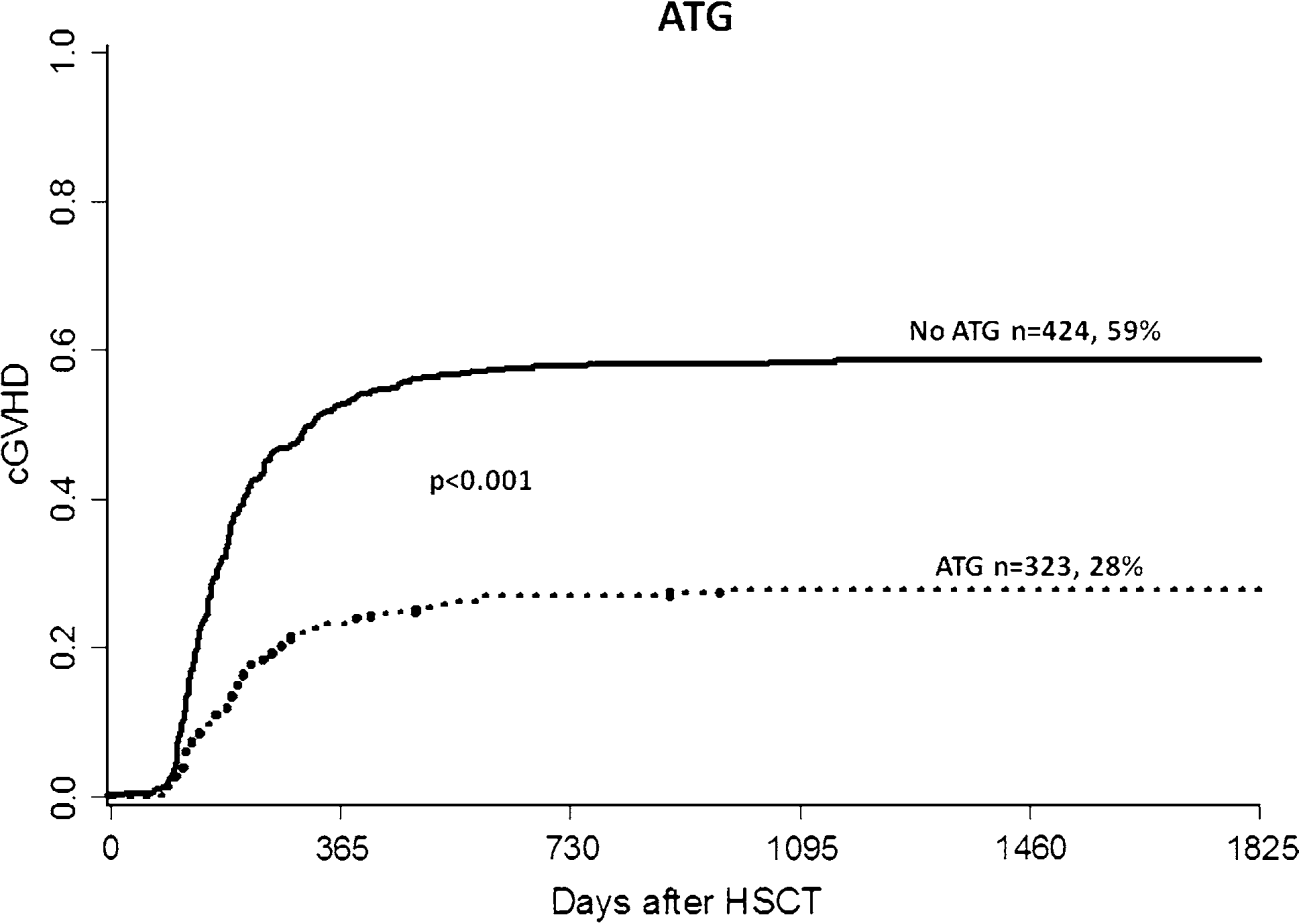
Chronic GVHD incidence in patients treated with anti-thymocyte globulin (ATG) versus no ATG

In univariate analysis, significant risk factors for the development of cGVHD included prior acute GVHD, RIC, PBSC, sex mismatch, increased donor age, sibling donor, and late disease stage (beyond first remission). The use of ATG was a protective factor (Fig. 1). In multivariate analysis, the following variables significantly influenced the risk of overall cGVHD: use of ATG [HR = 0.41, 95% CI (0.32–0.52), *p* < 0.001], higher patient age (in 10-year increments) [HR = 1.15, 95% CI (1.07–1.24), *p* < 0.001], prior acute GVHD [HR = 1.30, 95% CI (1.04–1.63), *p* = 0.024] and reduced intensity conditioning (RIC) [HR = 1.36, 95% CI (1.04–1.79), *p* = 0.028]. When analyzing risk factors for cGVHD after correcting for differences between patients receiving RIC or MAC, it is apparent that RIC patients still have higher cGVHD incidence [HR = 1.38, 95% CI (1.02–1.88), *p* = 0.038]. The analysis was done in order to eliminate confounding factors in each group.

### Risk factors for severe cGVHD

The overall incidence of severe cGVHD was 14% (95% CI 11–17%). In multivariate analysis, female donor to male recipient [HR 1.80 (95% CI 1.17–2.78), *p* = 0.008], RIC [HR 4.85 (95% CI 2.40–9.83) *p* < 0.001], and prior aGVHD [HR 2.14 (95% CI 1.34–3.42), *p* = 0.001] significantly increased the risk of severe cGVHD while ATG had a protective effect [HR 0.20, (95% CI 0.11–0.37), *p* < 0.001].

**Fig. 2:**
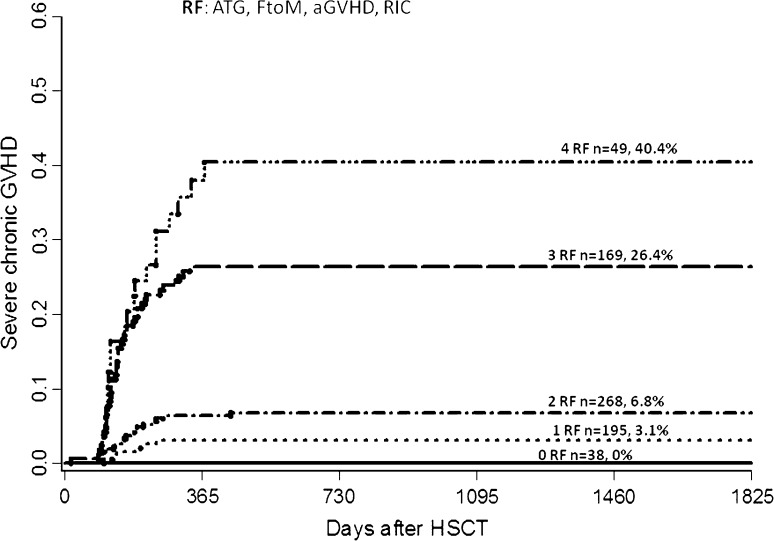
Risk factor score for developing severe cGVHD including risk factors from multivariate analysis with female donor to male recipient, reduced intensity conditioning (RIC), anti-thymocyte globulin (ATG) and prior acute GVHD

Based on our findings, we developed a scoring system including significant risk factors from multivariate analysis with regard to severe cGVHD incidence (Fig. 2). According to this scoring system, the risk of developing severe cGVHD was 3.1, 6.8, 26.4, and 40.4% at 5 years post-transplant among patients with one, two, three, or four risk factors.

Next, we conducted a multivariate analysis including only significant risk factors present at the time of transplantation. In this analysis, we found that patient age > 45 years, female-to-male donation, and RIC increased the risk of severe cGVHD, while ATG remained a protective factor. When two to three of these risk factors were present, there was a significant protective effect from ATG on the incidence of severe cGVHD at 5 years post-transplant (Table 2).

**Table 2:** Incidence of severe chronic GVHD after HSCT depending on number of risk factors (RF) and inclusion of anti-thymocyte globulin (ATG) or not in the conditioning therapy. Only factors known at time of transplantation were analyzed

Severe cGVHD	ATG (%)	No ATG (%)	*p* value
1 RF	4	6	NS
2 RF	4	24	< 0.001
3 RF	7	40	0.01

### Transplant-related mortality (TRM), overall, and relapse-free survival

Concerning OS, the worst outcome was seen in patients with severe cGVHD or no cGVHD. Similar results were observed concerning TRM. In this analysis, we found that the best outcomes were obtained in patients who developed mild–moderate cGVHD (Table 3).

**Table 3:** Overall survival (OS) and transplant-related mortality (TRM) 5 years after HSCT, depending on the severity of chronic GVHD (95% confidence interval given in brackets)

Grade of cGVHD	TRM	OS
No cGVHD	24% (19–29%)	51% (46–56%)
Mild	14% (8–20%)	72% (63–81%)
Moderate	18% (11–25%)	71% (63–79%)
Severe	31% (21–41%)	50% (39–61%)
*p* value	< 0.001	< 0.001
All patients	22% (19–25%)	57% (53–61%)

**Fig. 3:**
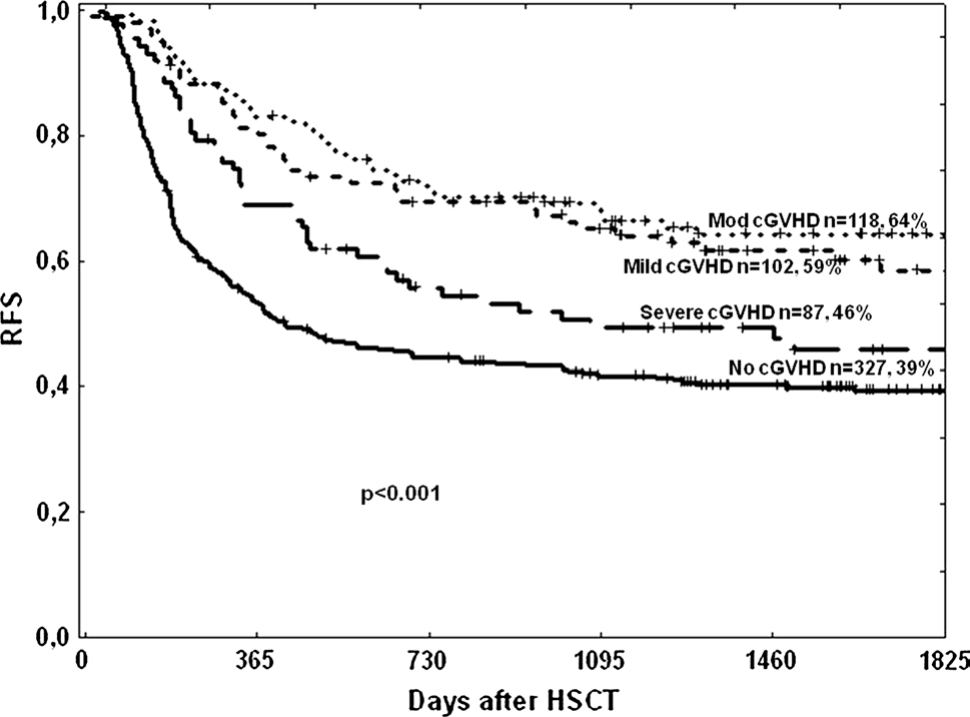
Relapse-free survival dependent on severity of chronic GVHD

**Fig. 4:**
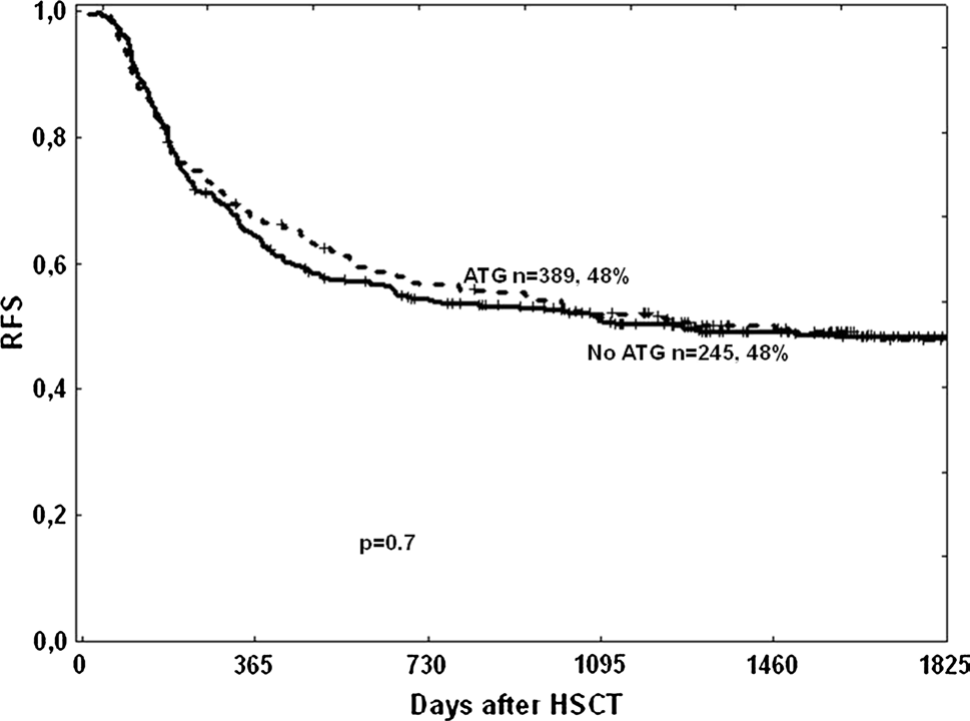
Relapse-free survival in patients conditioned with or without anti-thymocyte globulin (ATG)

Relapse-free survival at 5 years was similar in patients developing mild or moderate cGVHD [59% (49–59%) vs. 64% (55–73%), respectively], but significantly higher in these same groups compared to patients without cGVHD or with severe cGVHD [RFS of 39% (34–44%) and 46% (35–57%), respectively; *p* < 0.001 for mild and moderate compared to severe or no cGVHD] (Fig. 3). ATG had no influence on RFS (Fig. 4).

The risk of relapse or death was increased among patients with high-risk disease at transplantation [HR = 1.61, (95% CI 1.29–2.01), *p* < 0.001]. Interestingly, the best RFS was observed in patients with mild-to-moderate cGVHD irrespective of disease stage at time of transplantation (Table 4).

**Table 4:** Relapse-free survival (RFS) in different grades of chronic GVHD according to disease stage

Five-year RFS	Early disease (%)	Late disease (%)	*p* value
No cGVHD	45	36	0.09
Mild	72	48	0.016
Moderate	83	53	< 0.001
Severe	49	44	0.80

*Early stage* CR1/CP1, *Late stage* beyond CR1/CP1

## Discussion

Chronic GVHD remains the major cause of morbidity and mortality in long-term survivors after allogeneic stem cell transplantation. However, it is also correlated with a strong graft-versus-tumor effect. Thus, proper management of patients should be based on individualized strategies taking into account the notion that the absence of cGVHD might hamper relapse-free survival while severe cGVHD leads to increased mortality due to infectious complications and organ failure [15–18].

In the current study, we identified risk factors with regard to developing severe cGVHD, including female donor-to-male donor recipient, prior aGVHD and use of reduced intensity conditioning. While the first two risk factors have previously been described, the latter requires further clarification. In this regard, the finding of a higher risk of cGVHD among patients receiving RIC is somewhat surprising, since previous studies have not been able to show a difference in terms of cGVHD incidence depending on the type of conditioning regimen [19, 20]. Mechanisms involved in the development of acute and chronic GVHD are not entirely congruent. In this regard, cGVHD is not simply the end stage of acute GVHD [21]. In accordance with this hypothesis, use of RIC might, in fact, decrease the risk of acute and increase the risk of chronic GVHD. It could be speculated that acute GVHD is mostly dependent on the cytokine storm mediated by the tissue injury induced by the high doses of chemoradiotherapy, which is avoided in the RIC setting. On the other hand, chronic GVHD would be more dependent on the persistence of host–origin antigen-presenting cells that might trigger an alloresponse in donor T cells. The use of RIC favors the persistence of a mixed chimerism for a longer period post-transplant as compared to myeloablative conditioning. It could also be argued that since many centers show a preference toward treating older patients with RIC mainly due to comorbidities, the median age in this patient group is higher compared to those treated with myeloablative conditioning (MAC). This is also true in our study, and therefore, RIC patients are a higher-risk subpopulation for the development of cGVHD compared to MAC.

In the present study, we have shown that the addition of ATG decreases the overall incidence of cGVHD without hampering relapse-free survival, which is consistent with previous studies [6, 11, 17, 22–25].

Langston et al. showed that the addition of ATG to fludarabine–melphalan in the RIC regimen for patients receiving partially matched URD transplants does not increase the rates of relapse or infection. The same study did not show any difference with regards to cGVHD incidence in the two groups with Fu–Mel–ATG versus Flu–Mel [26]. One recent report shows that a fludarabine-containing RIC regimen with the addition of ATG is effective and safe for adults up to the age of 69 [27]. Investigation of the effect of ATG in a matched related donor myeloablative setting has shown beneficial effects with reduction in both severe aGVHD and cGVHD, which translated into higher survival [28].

Nevertheless, the use of pretransplant ATG as GVHD prophylaxis in patients receiving grafts from URD has been discussed for years, and although most studies have shown a significant reduction in acute GVHD and cGVHD, they have lacked evidence concerning a potential benefit on overall survival [11, 13, 24, 25, 29]. A GITMO group showed a significant reduction in the incidence of grades III–IV aGVHD and extensive cGVHD, but this positive effect was counterbalanced by the increased risk of infections. In a follow-up analysis of this study, encouraging results included a reduced risk of chronic lung dysfunction, protection against extensive chronic GVHD, and an improved quality of life [21]. In this prospective setting, there was no significant difference between the two groups regarding relapse and long-term survival.

These data contrast with a recent report from CIBMTR describing lower overall survival, lower disease-free survival, and an increased risk of relapse in patients receiving in vivo T-cell depletion [30]. This difference might be due to the heterogeneous nature of the population included in the CIBMTR study, in which patients received different preparations of ATG, dosage, conditioning regimens and donor types. In addition, the type of conditioning determines the effect of the antibody therapy since, according to Soiffer et al., there is a deleterious effect of in vivo T cell depletion among patients undergoing RIC. Furthermore, both the dosage and the preparation of ATG seem to be important in terms of transplant outcomes [13, 24, 25, 31]. In this regard, most studies have shown that an intermediate dose of rabbit-ATG (Thymoglobulin, Genzyme) ranging from 5 to 8 mg/kg leads to a positive reduction in severe aGVHD and cGVHD without impairing relapse or long-term survival.

In addition, in the current study, we show that increasing patient age significantly influences the incidence of cGVHD, but only up to 50 years, which is consistent with a recent study including 206 patients who underwent RIC allo-HSCT plus 5 mg/kg r-ATG. In this study, there was no difference in the incidence of extensive cGVHD in patient groups younger or older than 60 years [32].

Most importantly, the current study allowed us to develop a scoring system with clearly identifiable subgroups of patients at different risk of developing overall or severe cGVHD. Remarkably, both severe cGVHD and no cGVHD had a similar adverse impact on outcome. Based on these data, mild or moderate cGVHD show a desirable GvL effect without increasing the mortality from the procedure. Interestingly we found that in patients with more advanced disease stages the difference in RFS became less prominent, perhaps suggesting that this patient group relapses before a significant GvL effect is obtained. All together, these data suggest that patients at higher risk of severe cGVHD should be identified in order to have a favorable influence on long-term outcomes via the use of more intense immunosuppressive strategies, such as the use of ATG. In this regard, the lack of conclusive data regarding the impact of the use of ATG on survival in most previously reported randomized studies could be attributed to the lack of selection criteria, such that, according to our data, older males with female donors receiving RIC transplants would benefit the most from receiving this prophylaxis. Pretransplant identification of patients at risk of developing graft-versus-host disease remains an unmet medical need. In this regard, even among patients receiving transplantation from HLA-identical donors, the risk of GVHD varies greatly among individuals. Recently, Sorror et al. [33] reported that the pretransplant comorbidity index predicts the risk of severe aGVHD and subsequent mortality. In the current study, we developed a scoring system for cGVHD including risk factors known at the time of transplant: RIC, female-to-male donation and patient age > 45 years. Patients with these three risk factors had an incidence of severe cGHD of 40% at 5 years, while the same patients had a cumulative incidence of 7% at 5 years when they received ATG. Accordingly, the scoring system allows the tailoring of strategies to prevent cGVHD in the early post-transplant period and in the long-term follow-up. Furthermore, we also identified RIC, female-to-male donation, prior acute GVHD and not receiving ATG as GVHD prophylaxis as risk factors for developing severe cGVHD. This information would be of the greatest use for modifying immunosuppression during the post-transplant follow-up period. In this regard, these patients should be carefully monitored and might benefit from receiving early treatment once signs or symptoms of cGVHD appear.

In conclusion, in the current study, we identified subgroups of patients with different risk of cGVHD; these parameters might help to tailor GVHD prophylaxis and manage immunosuppression in the long term.
